# Educational attainment and sex modulate clinical outcomes in genetic frontotemporal dementia

**DOI:** 10.1093/braincomms/fcag262

**Published:** 2026-08-04

**Authors:** Enrico Premi, Damiano Archetti, Alberto Redolfi, Valeria Bracca, Valentina Cantoni, Armin Iraji, Vince D Calhoun, Sonia Bellini, Roberta Ghidoni, Roberto Gasparotti, Arabella Bouzigues, Lucy L Russell, Phoebe H Foster, Eve Ferry-Bolder, John C van Swieten, Lize C Jiskoot, Harro Seelaar, Raquel Sanchez-Valle, Robert Laforce, Caroline Graff, Daniela Galimberti, Rik Vandenberghe, Alexandre de Mendonça, Giuseppe Di Fede, Isabel Santana, Alexander Gerhard, Johannes Levin, Benedetta Nacmias, Markus Otto, Maxime Bertoux, Thibaud Lebouvier, Simon Ducharme, Christopher R Butler, Isabelle Le Ber, Elizabeth Finger, Maria Carmela Tartaglia, Mario Masellis, James B Rowe, Matthis Synofzik, Fermin Moreno, Henrik Zetterberg, Jonathan D Rohrer, Barbara Borroni, the GENetic Frontotemporal dementia Initiative (GENFI), Rhian Convery, Martina Bocchetta, David Cash, Sophie Goldsmith, Kiran Samra, David L Thomas, Thomas Cope, Maura Malpetti, Antonella Alberici, Emanuele Buratti, Andrea Arighi, Chiara Fenoglio, Vittoria Borracci, Maria Serpente, Tiziana Carandini, Emanuela Rotondo, Giacomina Rossi, Giorgio Giaccone, Pietro Tiraboschi, Paola Caroppo, Sara Prioni, Veronica Redaelli, David Tang-Wai, Ekaterina Rogaeva, Miguel Castelo-Branco, Morris Freedman, Ron Keren, Sandra Black, Sara Mitchell, Christen Shoesmith, Robert Bartha, Rosa Rademakers, Jackie Poos, Janne M Papma, Lucia Giannini, Liset de Boer, Julie de Houwer, Rick van Minkelen, Yolande Pijnenburg, Camilla Ferrari, Cristina Polito, Gemma Lombardi, Valentina Bessi, Enrico Fainardi, Stefano Chiti, Mattias Nilsson, Henrik Viklund, Melissa Taheri Rydell, Vesna Jelic, Abbe Ullgren, Elena Rodriguez-Vieitez, Tobias Langheinrich, Albert Lladó, Anna Antonell, Jaume Olives, Mircea Balasa, Nuria Bargalló, Sergi Borrego-Ecija, Ana Verdelho, Carolina Maruta, Tiago Costa-Coelho, Gabriel Miltenberger, Frederico Simões do Couto, Alazne Gabilondo, Ioana Croitoru, Mikel Tainta, Myriam Barandiaran, Patricia Alves, Benjamin Bender, David Mengel, Lisa Graf, Annick Vogels, Mathieu Vandenbulcke, Philip Van Damme, Rose Bruffaerts, Koen Poesen, Pedro Rosa-Neto, Maxime Montembeault, Raffaella Lara Migliaccio, Ninon Burgos, Daisy Rinaldi, Catharina Prix, Elisabeth Wlasich, Olivia Wagemann, Sonja Schönecker, Alexander Maximilian Bernhardt, Anna Stockbauer, Jolina Lombardi, Sarah Anderl-Straub, Adeline Rollin, Gregory Kuchcinski, Vincent Deramecourt, João Durães, Marisa Lima, Maria João Leitão, Maria Rosario Almeida, Miguel Tábuas-Pereira, Sónia Afonso, João Lemos

**Affiliations:** Stroke Unit, Azienda Socio Sanitaria Territoriale (ASST) Spedali Civili, Brescia 25123, Italy; Laboratory of Neuroinformatics, IRCCS Istituto Centro San Giovanni di Dio Fatebenefratelli, Brescia 25125, Italy; Laboratory of Neuroinformatics, IRCCS Istituto Centro San Giovanni di Dio Fatebenefratelli, Brescia 25125, Italy; Department of Clinical and Experimental Sciences, University of Brescia, Brescia 25123, Italy; Department of Molecular and Translational Medicine, University of Brescia, Brescia 25123, Italy; Department of Clinical and Experimental Sciences, University of Brescia, Brescia 25123, Italy; Tri-Institutional Center for Translational Research in Neuroimaging and Data Science (TReNDS), Georgia State, Georgia Tech and Emory, Atlanta, GA 30303, USA; Tri-Institutional Center for Translational Research in Neuroimaging and Data Science (TReNDS), Georgia State, Georgia Tech and Emory, Atlanta, GA 30303, USA; Molecular Markers Laboratory, IRCCS Istituto Centro San Giovanni di Dio, Fatebenefratelli, Brescia 25125, Italy; Molecular Markers Laboratory, IRCCS Istituto Centro San Giovanni di Dio, Fatebenefratelli, Brescia 25125, Italy; Neuroradiology Unit, University of Brescia, Brescia 25123, Italy; INSERM U1127, Paris Brain Institute, Sorbonne Université, Hôpital Pitié-Salpêtrière, Paris 75013, France; Dementia Research Centre, Department of Neurodegenerative Disease, UCL Queen Square Institute of Neurology, London WC1N 3AR, UK; Dementia Research Centre, Department of Neurodegenerative Disease, UCL Queen Square Institute of Neurology, London WC1N 3AR, UK; Dementia Research Centre, Department of Neurodegenerative Disease, UCL Queen Square Institute of Neurology, London WC1N 3AR, UK; Dementia Research Centre, Department of Neurodegenerative Disease, UCL Queen Square Institute of Neurology, London WC1N 3AR, UK; Department of Neurology, Erasmus Medical Centre, Rotterdam 3000 CA, Netherlands; Department of Neurology, Erasmus Medical Centre, Rotterdam 3000 CA, Netherlands; Department of Neurology, Erasmus Medical Centre, Rotterdam 3000 CA, Netherlands; Alzheimer’s Disease and Other Cognitive Disorders Unit, Neurology Service, Hospital Clínic, Institut D’Investigacións Biomèdiques August Pi I Sunyer, University of Barcelona, Barcelona 08036, Spain; Clinique Interdisciplinaire de Mémoire, Département des Sciences Neurologiques, CHU de Québec, Québec City, Canada G1J 1Z4; Faculté de Médecine, Université Laval, Quebec City, Canada G1Z 1J4; Department of Neurobiology, Care Sciences and Society, Center for Alzheimer Research, Division of Neurogeriatrics, Bioclinicum, Karolinska Institutet, Solna 171 64, Sweden; Unit for Hereditary Dementias, Theme Inflammation and Aging, Karolinska University Hospital, Solna 171 64, Sweden; Fondazione IRCCS Ca’ Granda, Dipartimento Area Neuroscienze e Salute Mentale, Ospedale Maggiore Policlinico, Milan 20122, Italy; Department of Biomedical, Surgical and Oral Sciences, University of Milan, Centro Dino Ferrari, Milan 20122, Italy; Laboratory for Cognitive Neurology, Department of Neurosciences, KU Leuven, Leuven 3000, Belgium; Neurology Service, University Hospitals Leuven, Leuven 3000, Belgium; Leuven Brain Institute, KU Leuven, Leuven 3000, Belgium; Faculty of Medicine, University of Lisbon, Lisbon 1649-028, Portugal; Dipartimento di Neuroscienze Cliniche, Fondazione IRCCS Istituto Neurologico Carlo Besta, Milan 20126, Italy; Neurology Service, Faculty of Medicine, University Hospital of Coimbra, University of Coimbra, Coimbra 3004-561, Portugal; Center for Neuroscience and Cell Biology, Faculty of Medicine, University of Coimbra, Coimbra 3004-504, Portugal; Division of Psychology Communication and Human Neuroscience, Wolfson Molecular Imaging Centre, University of Manchester, Manchester M20 3JJ, UK; Department of Nuclear Medicine, Center for Translational Neuro- and Behavioral Sciences, University Medicine Essen, Essen 45147, Germany; Department of Geriatric Medicine, Klinikum Hochsauerland, Arnsberg 59759, Germany; Department of Neurology, Ludwig-Maximilians Universität München, Munich 80336, Germany; Department of Neurology, German Center for Neurodegenerative Diseases (DZNE), Munich 81377, Germany; Munich Cluster of Systems Neurology (SyNergy), Munich 81377, Germany; Department of Neuroscience, Psychology, Drug Research and Child Health, University of Florence, Florence 50139, Italy; IRCCS Fondazione Don Carlo Gnocchi, Florence 50143, Italy; Department of Neurology, University of Ulm, Ulm 89081, Germany; Inserm, Lille Neuroscience & Cognition U1172, University of Lille, CHU Lille, Lille 5900, France; Inserm, Lille Neuroscience & Cognition U1172, University of Lille, CHU Lille, Lille 5900, France; Douglas Mental Health University Institute, Department of Psychiatry, McGill University Health Centre, McGill University, Montreal, Québec, Canada H4A 3J1; McConnell Brain Imaging Centre, Montreal Neurological Institute, McGill University, Montreal, Québec, Canada H3A 2B4; Nuffield Department of Clinical Neurosciences, Medical Sciences Division, University of Oxford, Oxford OX3 9DU, UK; Department of Brain Sciences, Imperial College London, London W12 0NN, UK; INSERM U1127, Paris Brain Institute, Sorbonne Université, Hôpital Pitié-Salpêtrière, Paris 75013, France; Centre de Référence des Démences Rares ou Précoces, IM2A, Département de Neurologie, AP-HP—Hôpital Pitié-Salpêtrière, Paris 75013, France; Département de Neurologie, AP-HP—Hôpital Pitié-Salpêtrière, Paris 75013, France; Department of Clinical Neurological Sciences, University of Western Ontario, London, Ontario, Canada N6A 5A5; Tanz Centre for Research in Neurodegenerative Diseases, University of Toronto, Toronto, Ontario, Canada M5T 0S8; Sunnybrook Health Sciences Centre, Sunnybrook Research Institute, University of Toronto, Toronto, Canada M4N 3M5; Department of Clinical Neurosciences and Cambridge University Hospitals NHS Trust, University of Cambridge, Cambridge CB2 0QQ, UK; Division Translational Genomics of Neurodegenerative Diseases, Hertie-Institute for Clinical Brain Research and Center of Neurology, University of Tübingen, Tübingen 72076, Germany; Center for Neurodegenerative Diseases (DZNE), Tübingen 72076, Germany; Cognitive Disorders Unit, Department of Neurology, Hospital Universitario Donostia, San Sebastian 20014, Spain; Neurosciences Area, Group of Neurodegenerative Diseases, Biogipuzkoa Health Research Institute, San Sebastian 20014, Spain; Center for Biomedical Research in Neurodegenerative Disease (CIBERNED), Carlos III Health Institute, Madrid 28031, Spain; Dementia Research Centre, Department of Neurodegenerative Disease, UCL Queen Square Institute of Neurology, London WC1N 3AR, UK; Department of Psychiatry and Neurochemistry, Institute of Neuroscience & Physiology, the Sahlgrenska Academy at the University of Gothenburg, Mölndal SE-43141, Sweden; Clinical Neurochemistry Laboratory, Sahlgrenska University Hospital, Mölndal SE-431 80, Sweden; Department of Neurodegenerative Disease, UCL Institute of Neurology, London WC1N 3BG, UK; Hong Kong Center for Neurodegenerative Diseases, Clear Water Bay 999077, Hong Kong, China; Wisconsin Alzheimer’s Disease Research Center, University of Wisconsin School of Medicine and Public Health, University of Wisconsin-Madison, Madison, WI 53792, USA; Dementia Research Centre, Department of Neurodegenerative Disease, UCL Queen Square Institute of Neurology, London WC1N 3AR, UK; Department of Clinical and Experimental Sciences, University of Brescia, Brescia 25123, Italy; Molecular Markers Laboratory, IRCCS Istituto Centro San Giovanni di Dio, Fatebenefratelli, Brescia 25125, Italy

**Keywords:** frontotemporal lobar degeneration, genetic, education, sex, Discriminative Event-Based Model (DEBM)

## Abstract

Individuals with autosomal dominant frontotemporal dementia (FTD) exhibit considerable variability in disease onset and progression. Both modifiable and non-modifiable factors—such as sex, educational attainment or geographic region of residence—may contribute to this heterogeneity, potentially through their influence on cognitive reserve. The aim of the present study was to investigate the role of cognitive reserve modulators within the Genetic Frontotemporal dementia Initiative (GENFI) cohort. To this end, we used functional MRI (i.e. spatial chronnectome measures) and neurodegenerative markers (i.e. plasma neurofilament light chains levels) to determine disease stage using a Discriminative Event-Based Model (DEBM). We then examined how potential modulators influence the relationship between disease stage and cognitive performance. We analysed a total of 711 participants, including 106 patients with genetic FTD, 325 presymptomatic mutation carriers and 280 non-carriers healthy controls. Female participants showed a weaker association between disease stage and cognitive performance compared to males (*P* < 0.001), with difference becoming progressively more pronounced across symptomatic stages. Educational attainment exhibited a similar effect: individuals with higher education demonstrated an attenuated association compared to those with secondary or primary schooling (*P* < 0.001), with differences already detectable at prodromal disease stages. The effect of geographical region of residence was associated with education levels, but appeared to have an indirect and less strong influence. In summary, sex and educational attainment significantly affect the development and maintenance of cognitive reserve in individuals with genetic FTD. These findings underscore the importance of identifying disease-modifying interventions since the presymptomatic stages of the disease.

## Graphical Abstract

**Graphical Abstract:**
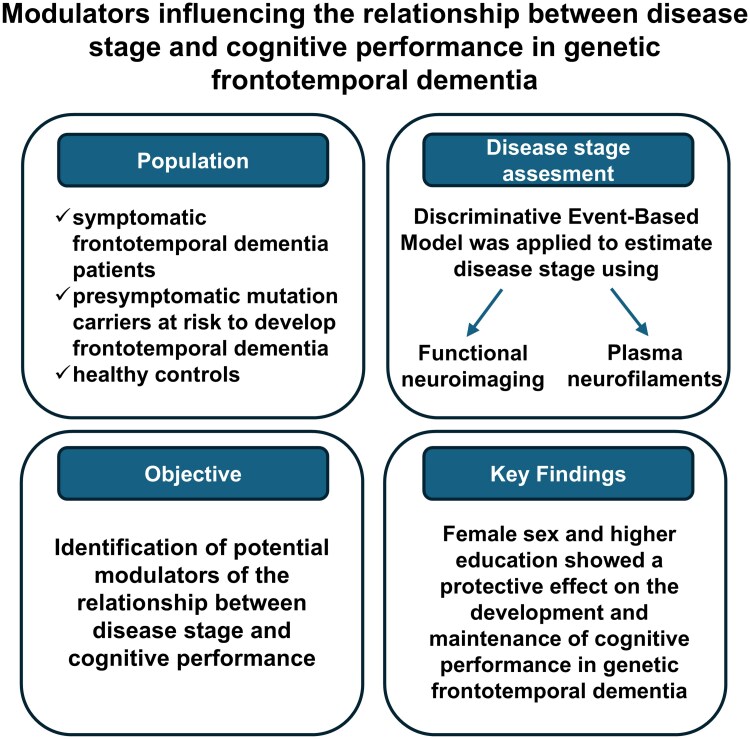

## Teaser

Premi and Archetti *et al*. studied cognitive reserve modulators in patients with autosomal dominant frontotemporal dementia using functional MRI and neurodegenerative markers to determine disease stage with a Discriminative Event-Based Model. They observed that sex and educational attainment significantly affect cognitive reserve.

## Introduction

Genetic frontotemporal dementia (FTD) encompasses a spectrum of heterogeneous neurodegenerative disorders, most commonly caused by an autosomal dominant mutations in the chromosome 9 open reading frame 72 (*C9orf72*), progranulin (*GRN*) or microtubule associated protein tau (*MAPT*) genes.^1^ Despite shared genetic origins, affected individuals often display substantial variability in age at onset and disease progression, even within the same mutation or family.^2^ Understanding the determinants of this variability could help identify potential targets for intervention, either to delay symptom onset in *at-risk* individuals or to slow disease progression in symptomatic patients.

In this context, cognitive reserve has emerged as a key framework for understanding heterogeneity in cognitive ageing, including FTD.^3-6^ Cognitive reserve refers to the ability to maintain cognitive function despite neuropathological damage, a concept used to explain heterogeneity in clinical outcomes among individuals with similar levels of brain pathology.^7^ Certain modifiable factors, most notably educational and occupational attainment, have been shown to enhance the capability to maintain cognitive performance relatively well in ageing and disease.^8-10^ Other factors, including sex and socio-environmental context, may also contribute to cognitive reserve. In this regard, it has been reported that females with genetic and sporadic FTD present higher cognitive reserve than males, suggesting that sex modulates resilience to frontotemporal neurodegeneration.^11,12^ Additionally, neighbourhood disadvantage has been associated with reduced cognitive reserve,^13^ but the role played by country or region of residence, which may reflect diverse cultural, social and environmental contexts, has never been explored in large multinational FTD cohorts.

Although these studies offer preliminary insights, a comprehensive evaluation of multiple modifiable and non-modifiable predictors, and their interactions, across the full disease course, from the presymptomatic to symptomatic disease stages, has never been conducted.

From a conceptual perspective, assessing how these modulating factors influence the relationship between brain disease stage and cognitive performance could provide valuable insight into the mechanisms underlying cognitive reserve, and inform the development of targeted, preventive strategies.

These observations prompted the present study, in which we used a data-driven model of disease progression,^14,15^ based on dynamic brain functional connectivity MRI and Neurofilament Light Chain (NfL) data, to investigate the role of educational attainment, sex and geographic region of residence as proxies for cognitive reserve in individuals with autosomal dominant FTD and *at-risk* individuals from the GENFI cohort. If these factors are found to influence cognitive reserve, they may have direct clinical implications for future preventive and therapeutic strategies for genetic FTD.

## Materials and methods

### Participants

Data for this study were drawn from the GENFI multicentre cohort study,^1^ which consists of research centres in the Europe, the UK and Canada (www.genfi.org.uk).

For this study, symptomatic and presymptomatic subjects carrying pathogenetic variants in *GRN, MAPT* or *C9orf72* genes were included. Non-carriers were included as healthy subjects. All participants underwent the GENFI standardized assessment.^16^ Demographic characteristics and country of residence were recorded for all participants. Local ethics committees approved the study at each site, and all participants provided written informed consent. The study was conducted according to the Declaration of Helsinki.

### Study design and hypothesis

**Figure 1:**
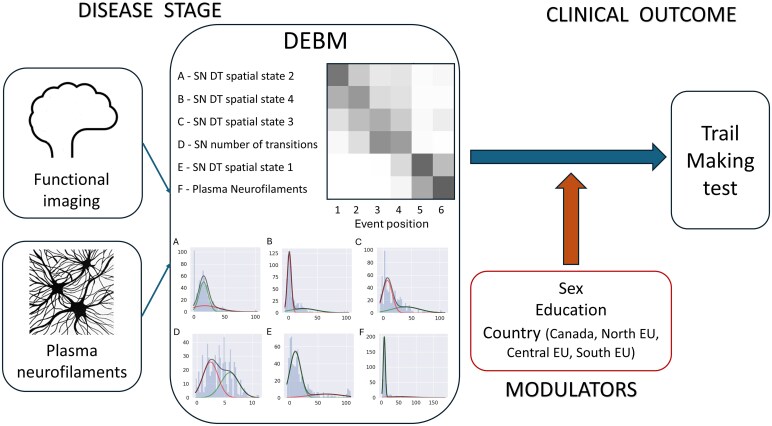
**Study design.** DEBM = Discriminative Event-Based Model; DT = Dwell Time; EU = European Union; SN = Salience Network. A data-driven Discriminative Event-Based Model that allows for patient staging was assessed through resting state functional imaging measures and plasma neurofilament levels. Clinical performances were evaluated by Trail Making Test part A (TMT A) and part B (TMT B). Sex, educational level and country of residence were examined as potential modulators of the relationship between disease stage and clinical performances.

The study aimed to investigate the influence of potentially modifiable and non-modifiable factors on the relationship between disease stage and clinical outcomes. As reported in Fig. 1, a data-driven disease progression model that allows for patient staging was assessed through brain MRI and NfL levels. Regarding brain MRI data, resting state functional imaging measures were used to compute the spatial chronnectome (see below). Clinical performances were evaluated by Trail Making test part A and part B (TMT A and TMT B), which provides a measure of processing speed, visual-conceptual tracking, mental flexibility and executive functioning.

The following variables were examined as potential modulators of the relationship between disease stage and clinical performances: (i) sex; (ii) level of schooling (education), i.e. primary (up to 9 years of education), secondary (between 10 and 12 years of education), higher (more than 13 years of education); (iii) country of residence, i.e. Northern Europe (Sweden and UK), Central Europe (Germany, Netherlands, Belgium, France), Southern Europe (Spain, Portugal and Italy) and Canada.

### Neurofilament light quantification

Plasma samples was collected according to standard procedures, as previously published.^17^ NfL levels were measured in duplicates by the single molecule array (Simoa) technique on the Simoa HD-X Analyzer (Quanterix, Lexington, MA, USA), using the NF-light Advantage kit for NfL,^17^ according to the manufacturer’s instructions (dilution: 1/4). All records have a coefficient of variation (CV) < 20%. Technicians were blinded to clinical diagnosis or mutation status.

### MRI acquisition and preprocessing

The MRI protocol was standardized across all GENFI sites and adapted for different scanners; no pre-study phantom harmonization was performed at the local level. This study used T2-weighted echo-planar imaging (EPI) sequences sensitized to blood oxygenation level-dependent (BOLD) contrast for resting state-fMRI (considering that EPI normalization is able to reduce variability across subjects potentially amplifying the power of the sample).^18^ During scanning, participants were instructed to keep their eyes closed, avoid thinking about anything specific and refrain from falling asleep. Functional data were pre-processed using the toolbox for Data Processing & Analysis for Brain Imaging (DPABI, http://rfmri.org/dpabi)^19^ based on Statistical Parametric Mapping (SPM12, https://www.fil.ion.ucl.ac.uk/spm/). The preprocessing pipeline follows the approach previously described by Premi *et al*.^20^ Differences in repetition times (TRs), which ranged from 2200 to 2500 μs, were taken into account during preprocessing [for Independent component analysis (ICA)] and dynamic functional connectivity (i.e. spatial chronnectome analysis). To mitigate head motion during MRI, a framewise displacement (FD) threshold of less than 0.5 was used in line with previous literature data on the impact of head motion on dynamic functional network connectivity.^20-22^ For each participant, the first five volumes of the fMRI series were discarded to account for magnetization equilibration. The remaining volumes underwent slice-timing correction and were realigned to the first volume. Subjects that had a maximum displacement in any direction larger than 3.0 mm, or a maximum rotation (*x,y,z*) larger than 3.0°, was excluded from the analysis. The number of volumes varied across the GENFI centres, ranging from 140 to 200. For harmonization, a total of 603 patients with EPI series with volumes were cropped to the first 135 volumes prior to ICA decomposition (mean acquisition time 322.95 ± 19.37 s).

### Functional networks decomposition and spatial chronnectome analysis

Functional imaging data were processed with GIFT toolbox (http://trendscenter.org/software/gift).^23^ We applied a spatially constrained multivariate objective optimization ICA with reference (MOO-ICAR)^24,25^ to extract 12 spatial maps of large-scale brain networks.^26^ For the purpose of this study, we selected the Salience Network (SN) as our network of interest because primarily affected in FTD (see details in Ref^20^.

The spatial chronnectome analysis was conducted using the dynamic FNC toolbox implemented in GIFT toolbox (http://trendscenter.org/software/gift). For each subject, the dynamic component maps (dCMs) of SN were computed using a sliding window approach and then clustered via *k*-means into a discrete set of spatial states.^20^ The number of spatial states was set to 4.^26^ Specifically, *k*-means clustering was applied on the 76 788 dCMs (711 subjects × 108 windows) of the brain network, and it was repeated 100 times with different initializations, as previously published.^20^ State-level dynamic metrics were calculated for the SN, in particular the mean dwell time (DT), that is the average of the amount of time that subjects spend in a state before transitioning, and the number of transitions (NT), that is the total number of transitions among SN states performed by subjects. The four spatial states of the SN were characterized by a frontal hub, with a more pronounced signal in the insula region, bilaterally. States 1, 2 and 3 showed an additional anticorrelation with frontoparietal and temporal regions (see Supplementary Fig. 1).

### Disease progression modelling

The disease progression was modelled by means of a Discriminative Event-Based Model (DEBM)^27^ which is a modelling approach based on the fundamental works by Fonteijn *et al*.^14^ and Young *et al*.^15^ on event-based modelling. According to this approach, each biomarker, namely the DT of the four SN spatial states, the NT between the SN spatial states, and the plasma NfL, can assume either the status of ‘normal’ or ‘abnormal’, and the probabilistic transition from the normal to the abnormal state is defined as an event. The aim is to define, in a data-driven manner, the sequence of events that describe the most probable cascade of biological changes that characterizes the transition of a subject from the healthy condition to the diseased state.

The probability of an event for each biomarker is determined by a Gaussian mixture model (GMM) in which the normal and abnormal components are modelled by Gaussian distributions, with the initial distributions estimated based on data from healthy and symptomatic participants, respectively, as defined by a Bayesian classifier trained to remove outliers and wrongly labelled data. The initial distributions are then refined using data from all participants by a GMM that optimizes the distribution parameters alternately between the Gaussian parameters and the mixture parameters until the latter converge.

The calculation of the event ordering is a two-step process where first (i) a specific ordering is calculated for each participant by sorting the posterior probability that each biomarker has become abnormal and then (ii) the central ordering is calculated as the event sequence that minimizes the sum of the probabilistic Kendall’s tau distances^27^ between itself and all the subject-wise orderings. Since the posterior probability is influenced by the physiological variability of the biomarkers, DEBM assumes that the ordering of each participant is a noisy estimate of the central ordering.

The optimal sequence was calculated as the average of event orderings from 500 bootstrapped iterations of DEBM and served as a staging sequence where each participant can be placed on the *k*-th step of the sequence that maximizes the probability that all events up to *k* have already occurred and events from *k*+1 to the end of the sequence are yet to occur.

Balanced accuracy at optimal threshold *k_T_* was calculated for pairwise comparisons between FTD patients versus presymptomatic mutation carriers and presymptomatic mutation carriers versus healthy subjects to define sections of the central ordering that correspond to each diagnostic class.

### Investigation of group differences and statistical analysis

To investigate how modulators influence the relationship between biological disease stage and clinical outcome, TMT A, TMT B and TMT B-A scores (corrected for the effects of age by means of linear regression with the group of healthy subjects as reference^28^) were regressed against disease staging from specific levels defined for each covariate. All modulators were initially studied independently and differences among levels were assessed using the ANCOVA test. The ANCOVA test was performed for each pair of levels. Given the possibility that correlations between covariates may confound the results of the regression, we performed Chi-Squared tests between each pair of covariates to check whether participants were uniformly distributed, and we also computed the effect size for each pair of covariates to assess their correlation by means of Cramér’s V.

The individual contribution of each modulator was also assessed by building complete interaction linear models that have disease staging and all modulators as independent variables and clinical outcomes as dependent variable (one interaction model for each clinical outcome). Then, we modified the model by removing one of the variables and assessed how the model degraded in terms of residual sum of squares (RSS). We assumed that the variable that most degraded the fit when removed was the most influent covariate on the relationship between biological disease progression and clinical outcome. Relevant differences between models were assessed with likelihood ratio tests. Significance level for each of the statistical tests was set at α = 0.05.

Disease modelling and all statistical analyses were performed with python version 3.10. Specifically, we employed python module pyebm (https://github.com/EuroPOND/pyebm) for disease stage modelling Python modules statsmodels 0.14.4 and scipy 1.15.2 for statistical analyses.

## Supplementary Material

fcag262_Supplementary_Data

## Figures and Tables

**Table 1:** Demographic and clinical characteristics of GENFI participants included in the present study

	All sample *n* = 711	HC *n* = 280	Presymptomatic *n* = 325	Symptomatic *n* = 106	*P*-value^c^
**Age, years**	48 ± 13	46 ± 13 ^a1,b^	45 ± 12 ^a1,b1^	61.7 ± 8.4 ^a^	0.04
**Sex, females % (*n*)**	56.1 (399)	56.8 (159)^a^	61.8 (201)^a^	36.8 (39)^a1^	<0.001
**Region of residence, % (*n*)**					
North Europe	21.4 (152)	19.6 (55)	22.2 (72)	23.6 (25)	0.56
Central Europe	28.6 (203)	26.8 (75)	29.8 (97)	29.2 (31)	0.65
South Europe	33.2 (236)	33.9 (95)	33.2 (108)	31.1 (33)	0.94
Canada	16.3 (116)	19.6 (55)	14.5 (47)	13.2 (14)	0.17
**Education, % (*n*)**					
Primary	8.9 (63)	8.9 (25)	7.4 (24)	13.2 (14)	0.18
Secondary	20.7 (147)	21.1 (59)	17.5 (57)^a1^	29.2 (31)^a^	0.01
Higher	70.5 (501)	70.0 (196)	75.1 (244)^a^	57.5 (61)^a1^	<0.001
**Genetic group, % (*n*)**					
*C9orf72*	45.2 (195)	-	40.6 (132) ^a1^	59.4 (63) ^a^	0.01
*GRN*	37.1 (160)	-	42.2 (137) ^a^	21.7 (23) ^a1^	<0.001
*MAPT*	17.6 (76)	-	17.2 (56)	18.9 (20)	0.81
**CDR® plus NACC FTLD** ^a,b^	0.0 (0.0–0.5)	0.0 (0.0–0.0)^a1^	0.0 (0.0–0.0)^a1^	2.00 (1.00–2.00)^a^	<0.001
**TMT A (s)** ^c^	33 ± 22	28 ± 11^a1^	29 ± 11^a1^	58 ± 37^a^	<0.001
**TMT B (s)** ^c^	81 ± 60	67 ± 32^a1^	70 ± 33^a1^	169 ± 90^a^	<0.001
**TMT B-A (s)** ^c^	50 ± 49	39 ± 28^a1^	41 ± 29^a1^	119 ± 75^a^	<0.001
**Plasma NfL (pg/ml)**	15.2 ± 21.2	9.7 ± 9.1^a1^	11.3 ± 15.0^a1^	49.3 ± 35.4^a^	<0.001

*C9orf72* = chromosome 9 open reading frame 72 expansion; *GRN* = progranulin mutation; *MAPT* = microtubule associated protein tau mutation; CDR plus NACC FTLD = CDR Dementia Staging Instrument PLUS National Alzheimer’s Coordinating Center (NACC) Frontotemporal Lobar Degeneration global scores; TMT A = Trail Making Test version A; TMT B = Trail Making Test version B; TMT B-A = TMT B scores minus TMT A scores; NfL = neurofilament light chain. Post hoc pairwise comparisons: ^a^ > ^a1^; and ^b^ > ^b1^. Kruskal Wallis, Chi-square test, or ANOVA were used for the analysis, with Bonferroni correction for multiple comparisons. Results are expressed as mean ± standard deviation, unless otherwise specified; ^a^ median (range); ^b^ 27 missing data; ^c^ corrected for age.

**Figure 2:**
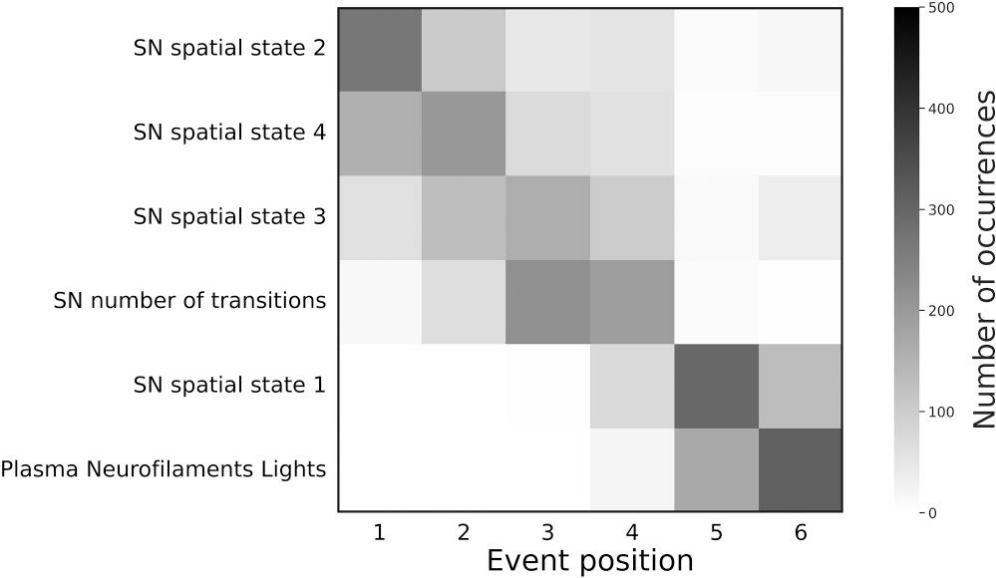
**Positional variance diagrams of event ordering obtained with Discriminative Event-Based Model (DEBM).** SN = Salience Network. Event ordering generated via the Discriminative Event-Based Model (DEBM) algorithm. The greyscale indicates how many times an event was placed at a specific position across the 500 bootstrapped iterations, with darker tones indicating a more consistent positioning of the event (*N* = 711).

**Figure 3:**
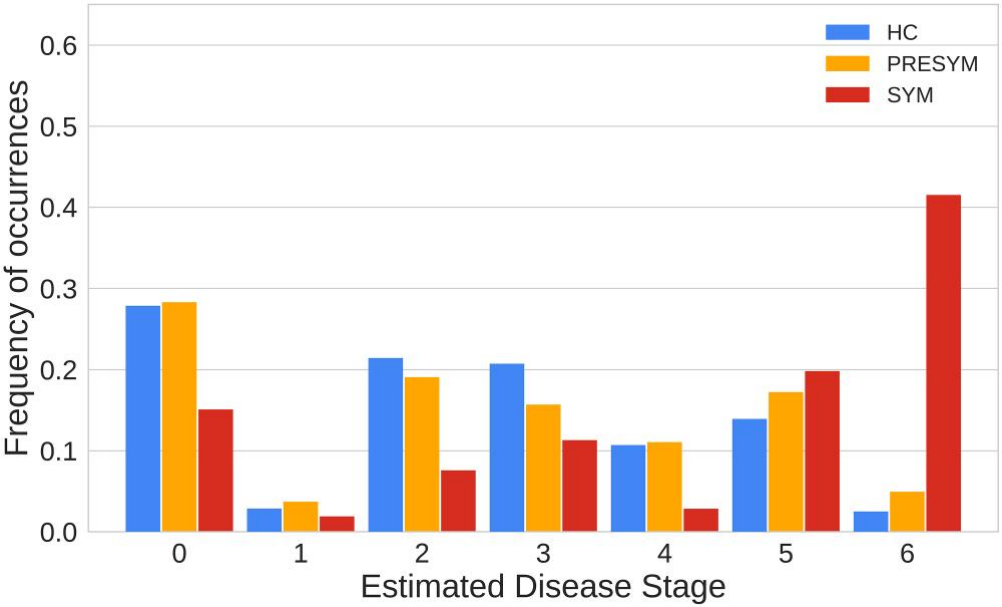
**Subjects’ disease stage based on the sequencing obtained with discriminative event-based model (DEBM).** Staging of subjects from all diagnostic categories (HC = healthy controls; Presym = presymptomatic subjects, Sym = FTD patients) are reported. Participants were assigned a disease stage based on the Discriminative Event-Based Model (DEBM) (*N* = 711). Histograms are normalized for each diagnostic category (group numerosity: HC = [stage0: 78, stage1: 8, stage2: 60, stage3: 58, stage4: 30, stage5: 39, stage6: 7]; Presym = [stage0: 92, stage1: 12, stage2: 62, stage3: 51, stage4: 36, stage5: 56, stage6: 16]; Sym = [stage0: 16, stage1: 2, stage2: 8, stage3: 12, stage4: 3, stage5: 21, stage6: 44]).

**Figure 4:**
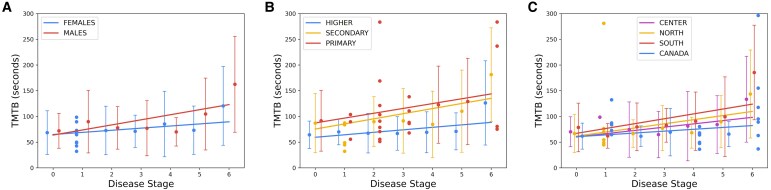
**Sex, educational attainment and country of residence moderate the relationship between stage and clinical outcomes in autosomal dominant FTD mutation carriers.** TMTB = Trail Making Test part B. Results of the ANCOVA between biological disease stage and clinical outcome (TMT B scores). A = Female participants showed a significantly attenuated association between disease stage and clinical performance, as measured by TMT B, compared to males (*F* = 11.6, *P* < 0.001). (*N* = 711). B = Individuals with higher education showed a weaker association between disease stage and cognitive decline than those with primary or secondary education (*F* = 29.2, *P* < 0.001). (*N* = 711). C = Country of residence had a modest but significant effect, with a weaker stage-performance association observed in participants residing in Canada and Central Europe (*F* = 4.55, *P* = 0.04) (*N* = 707). Circular dots represent the class average at a specific stage, bands represent standard deviation. If less than 10 participants are available at a specific stage standard deviation is not shown. Panel **A** group numerosity: Females = [stage0: 93, stage1: 9, stage2: 84, stage3: 67, stage4: 46, stage5: 70, stage6: 30]; Males = [stage0: 93, stage1: 13, stage2: 46, stage3: 54, stage4: 23, stage5: 46, stage6: 37]. Panel **B** group numerosity: Higher = [stage0: 137, stage1: 13, stage2: 99, stage3: 87, stage4: 47, stage5: 78, stage6: 40]: Secondary = [stage0: 35, stage1: 6, stage2: 22, stage3: 28, stage4: 11, stage5: 25, stage6: 20]; primary = [stage0: 14, stage1: 3, stage2: 9, stage3: 6, stage4: 11, stage5: 13, stage6: 7]. Panel **C** group numerosity: Centre = [stage0: 25, stage1: 1, stage2: 44, stage3: 32, stage4: 22, stage5: 59, stage6: 20]; North = [stage0: 55, stage1: 6, stage2: 20, stage3: 29, stage4: 13, stage5: 13, stage6: 16]; South = [stage0: 66, stage1: 10, stage2: 51, stage3: 33, stage4: 27, stage5: 29, stage6: 20]; Canada = [stage0: 40, stage1: 5, stage2: 13, stage3: 27, stage4: 7, stage5: 15, stage6: 9].

**Table 2:** Effects of different modulators on the correlations between disease stage and cognitive performances

Variable	Stage		
	TMT A	TMT B	TMT B-A
***Sex, male* versus *female***	**0**.**007**	**<0**.**001**	**0**.**002**
***Region of residence***	**0**.**03**	**0**.**004**	**0**.**008**
Central EU versus Canada	0.31	0.27	0.12
Canada versus Norther EU	0.21	0.054	0.51
Central EU versus Southern EU	**0**.**03**	**<0**.**001**	**0**.**005**
Central EU versus Northern EU	0.74	0.32	0.27
Southern EU versus Northern EU	0.11	0.13	0.16
Canada versus Southern EU	**0**.**02**	**0**.**001**	**0**.**004**
***Educational attainment***	**<0**.**001**	**<0**.**001**	**<0**.**001**
Primary versus secondary	0.21	0.36	0.39
Primary versus higher	**<0**.**001**	**<0**.**001**	**<0**.**001**
Secondary versus higher	**<0**.**001**	**<0**.**001**	**<0**.**001**

TMT A = Trail Making Test version A; TMT B = Trail Making Test version B; TMT B-A = TMT B scores minus TMT A scores; EU = European Union. The table reports *P*-values of ANCOVA tests performed for each pair of covariate levels. *P*-values reported in bold indicate significant differences between levels.

**Table 3:** Individual contributions of covariates

Variable	TMT A		TMT B		TMT B-A	
	RSS diff %	*P-value*	RSS diff %	*P-values*	RSS diff %	*P-value*
Sex removed	1.6	0.004	2.8	<0.001	2.7	0.002
Region of residence removed	1.7	0.29	2.3	0.12	2.0	0.19
Educational attainment removed	5.9	<0.001	8.9	<0.001	8.0	<0.001

TMT A = Trail Making Test version A; TMT B = Trail Making Test version B; TMT B-A = TMT B scores minus TMT A scores; RSS diff = differences of residual sum of squares.

## Data Availability

The raw data of this project is part of GENFI. De-identified participant data can be accessed on reasonable request to the corresponding author and genfi@ucl.ac.uk.

## Results

### Participants

The present cohort included 711 participants, namely 106 FTD patients (63 with *C9orf72* expansion, 23 with *GRN* mutations and 20 with *MAPT* mutations), 325 presymptomatic mutation carriers (132 with *C9orf72* expansion, 137 with *GRN* mutations and 56 with *MAPT* mutations) and 280 healthy controls (HCs). Demographic and clinical characteristics and plasma NfL dosages are reported in Table 1.

### Disease stage assessment by functional MRI and plasma NfL data

Events ordering and staging of GENFI cohort’s subjects were computed. Central event sequences and their associated variances were generated from functional MRI metrics—specifically, SN dwell time (DT) of states 1–4, SN numbers of transitions (NT) and plasma Nfl levels—and were plotted as positional variance diagrams (see Fig. 2). Event ordering generated via the DEBM algorithm indicated that abnormalities in MRI-derived metrics preceded increases in plasma NfL concentrations.

As reported in Fig. 3, participants were assigned a disease stage based on the event sequences. Most HC were staged between position 0 and 3, while the majority of symptomatic subjects were clustered at stages 5 and 6. Presymptomatic subjects were spread across stages 1–5.

### Effect of modulators on the correlation between disease stage and cognitive performances

We investigated the modulation effect of sex, education and geographical region of residence. In line with the cognitive reserve framework,^7,8^ a weaker correlation between disease stage and cognitive performances was interpreted as indicative of a greater cognitive reserve.

Female participants showed a significantly attenuated association between disease stage and clinical performance, as measured by TMT B, compared to males (*F* = 11.6, *P* < 0.001). This sex-related difference increased progressively across disease stages, with no difference observed at stage 0 and the largest divergences occurring at stages 5 and 6 (Fig. 4, panel A). Comparable patterns were observed when cognitive performances were measured with TMT A or the difference score TMT B-A (Table 2 and Supplementary Fig. 2).

Educational attainment was also a significant modulator: individuals with higher education showed a weaker association between disease stage and cognitive decline than those with primary or secondary education (*F* = 29.2, *P* < 0.001). This effect was evident from stage 0 and increased steadily up to stage 6 (Fig. 4, panel B). Similar findings were replicated when cognitive performances were measured by TMT A or TMT B-A (Table 2 and Supplementary Fig. 2).

Country of residence had a modest but significant effect, with a weaker stage-performance association observed in participants residing in Canada and Central Europe (*F* = 4.55, *P* = 0.04) (Fig. 4, panel C). Again, similar results were found when using TMT A and TMT B–A (Table 2 and Supplementary Fig. 2).

All three mutations groups (*C9orf72, GRN, MAPT*) showed a similar TMT A, TMT B and TMT B-A trends, and the main modulator for each mutation remained educational attainment.

## Discussion

In the present study, we investigated the moderating effects of educational attainment, sex and geographic region of residence on cognitive performance in individuals with autosomal dominant FTD from presymptomatic to symptomatic disease stages.

Our data suggest that these factors may help preserve or support cognition by modulating the relationship between disease stage—as assessed by functional MRI connectivity alterations and blood-based markers of neurodegeneration—and cognitive outcomes.

To model disease progression, we employed the Discriminative Event-Based Model (DEBM), a data-driven approach that allows for the staging of individuals along a continuous disease trajectory. This method enables the integration of multiple biomarkers without prior assumptions about sequence or thresholds, making it particularly suitable for the study of genetically defined dementia that affects multiple domains, under the assumption that the disease progression pattern is the same across all individuals.^14,15,27^

In this large cohort of subjects with genetic FTD, the educational attainment emerged as a key factor explaining discrepancies between brain pathology and cognition, thereby supporting the concept of cognitive and/or brain reserve. Consistent with the findings from other neurodegenerative disorders,^29^ higher education may potentially modify cognitive deterioration also in genetic FTD. Our results confirm and extend previous evidence from genetic dementia cohorts such as the DIAN study and Columbian kindred carrying the PSEN1 E280A mutation, where higher educational attainment was associated with delayed estimated clinical onset.^30^ This is also in line with data described in genetic FTD supporting that an active lifestyle was associated with less functional decline and outperformed brain volume.^31^

We are extending these findings into a larger sample of FTD with autosomal dominant inheritance by demonstrating that cognitive reserve operates from the earliest presymptomatic stages of the disease. Individuals with higher level of education were able to tolerate greater brain changes/neurodegeneration process before reaching cognitive impairment levels comparable to those with lower educational backgrounds. Interestingly, educational attainment modulated cognitive decline similarly across genetic FTD groups, with higher levels of education associated with slower cognitive decline, consistent with previous works.^9,16^

It is notable that our results also support the existence of sex-related differences in the ability to cope with frontotemporal neurodegeneration, adding to a growing body of literature the importance of considering sex in the design of research protocols and clinical trials. Specifically, females carrying FTD-related mutations have higher reserve than males. Notably, this advantage does not appear to reflect premorbid cognitive differences but becomes evident in the early symptomatic disease phase and increases over time. While previous work has proposed sex as modulators of executive functions in FTD,^32^ the underlying mechanisms remain unclear. Notable differences have been described according to sex in mechanisms known to play a role in neurodegeneration, like neuroimmune processes and responses.^33^ Supporting this, an X-linked single nucleotide polymorphism has been shown to modulate the age of onset in carriers of *C9orf72* hexanucleotide expansion.^34^

We finally examined the role of geographical region of residence as potential proxy of cognitive reserve. Motivated by recent findings on different regional trajectories of survival among FTD patients,^35^ we sought to determine whether the association between living in Northern, Central or Southern Europe or Canada and cognition can be attribute to cognitive reserve. We indeed found that patients living in Canada and Central Europe showed a slight advantage, which may be due to a range of unmeasured variables such as socio-economic and environmental exposures. However, in multivariate models where we systematically excluded one variable at a time (education, sex or country of residence), the association between disease stage and cognitive performance remained significantly explained by education and sex, but not by country of residence. This suggests that the effect of country of residence on cognitive reserve is probably mediated by more direct factors, such as education level.

Several limitations of this study must be acknowledged. First, years of education represent only one aspect of cognitive reserve. Other contributors, such as lifestyle activities and occupation, were not assessed due to the retrospective design of the study. Second, clinical heterogeneity within genetic FTD, which includes behavioural variant FTD or primary progressive aphasia syndromes, may impact on selected cognitive outcomes. A limiting methodological factor for this study is the imbalance and heterogeneity across diagnostic classes in the GENFI data, for which mitigation strategies were applied. Also, the absence of an independent dataset to validate the progression model and the modulators effects may limit the generalizability of our results. Another methodological limitation is the DEBM assumption that disease progression is valid for all individuals. Future work could investigate the possibility of multiple disease progressions^36,37^ that may be associated with specific FTD mutations as well as for sporadic FTD patients. Furthermore, Trail Making test, which is the commonly used instrument for measuring cognitive impairment in FTD,^28^ cannot replace detailed neuropsychological testing and does not represent all aspects of cognitive decline. Moreover, while interactions between factors (*e.g.* sex, education level, and geographical region) are of significant interest, they were not analyzed due to the limited sample size. Such stratification would have critically undermined the statistical power of the study.

In conclusion, our findings support a potentially protective role of both modifiable (*e.g*. education) and non-modifiable (*e.g*. sex) factors in autosomal dominant FTD. These results call for further research into disease-modifying interventions that could be implemented since the presymptomatic stages to mitigate the impact of ongoing neurodegeneration.

## Appendix GENFI Consortium members.

Rhian Convery, Martina Bocchetta, David Cash, Sophie Goldsmith, Kiran Samra, David L. Thomas, Thomas Cope, Maura Malpetti, Antonella Alberici, Emanuele Buratti, Andrea Arighi, Chiara Fenoglio, Vittoria Borracci, Maria Serpente, Tiziana Carandini, Emanuela Rotondo, Giacomina Rossi, Giorgio Giaccone, Pietro Tiraboschi, Paola Caroppo, Sara Prioni, Veronica Redaelli, David Tang-Wai, Ekaterina Rogaeva, Miguel Castelo-Branco, Morris Freedman, Ron Keren, Sandra Black, Sara Mitchell, Christen Shoesmith, Robert Bartha, Rosa Rademakers, Jackie Poos, Janne M. Papma, Lucia Giannini, Liset de Boer, Julie de Houwer, Rick van Minkelen, Yolande Pijnenburg, Camilla Ferrari, Cristina Polito, Gemma Lombardi, Valentina Bessi, Enrico Fainardi, Stefano Chiti, Mattias Nilsson, Henrik Viklund, Melissa Taheri Rydell, Vesna Jelic, Abbe Ullgren, Elena Rodriguez-Vieitez, Tobias Langheinrich, Albert Lladó, Anna Antonell, Jaume Olives, Mircea Balasa, Nuria Bargalló, Sergi Borrego-Ecija, Ana Verdelho, Carolina Maruta, Tiago Costa-Coelho, Gabriel Miltenberger, Frederico Simões do Couto, Alazne Gabilondo, Ioana Croitoru, Mikel Tainta, Myriam Barandiaran, Patricia Alves, Benjamin Bender, David Mengel, Lisa Graf, Annick Vogels, Mathieu Vandenbulcke, Philip Van Damme, Rose Bruffaerts, Koen Poesen, Pedro Rosa-Neto, Maxime Montembeault, Raffaella Lara Migliaccio, Ninon Burgos, Daisy Rinaldi, Catharina Prix, Elisabeth Wlasich, Olivia Wagemann, Sonja Schönecker, Alexander Maximilian Bernhardt, Anna Stockbauer, Jolina Lombardi, Sarah Anderl-Straub, Adeline Rollin, Gregory Kuchcinski, Vincent Deramecourt, João Durães, Marisa Lima, Maria João Leitão, Maria Rosario Almeida, Miguel Tábuas-Pereira, Sónia Afonso and João Lemos.
