# Supplementary material for: Educational attainment and sex modulate clinical outcomes in genetic frontotemporal dementia
Source: Brain Commun. 2026 Aug 4;8(4):fcag262. doi: 10.1093/braincomms/fcag262 (PMC13435617; doi:10.1093/braincomms/fcag262)
Supplement: Supplementary file 1 [file fcag262_supplementary_data.pdf]

Supplementary Figure 1. The four spatial states of salience network as derived from spatial chronnectome analysis.

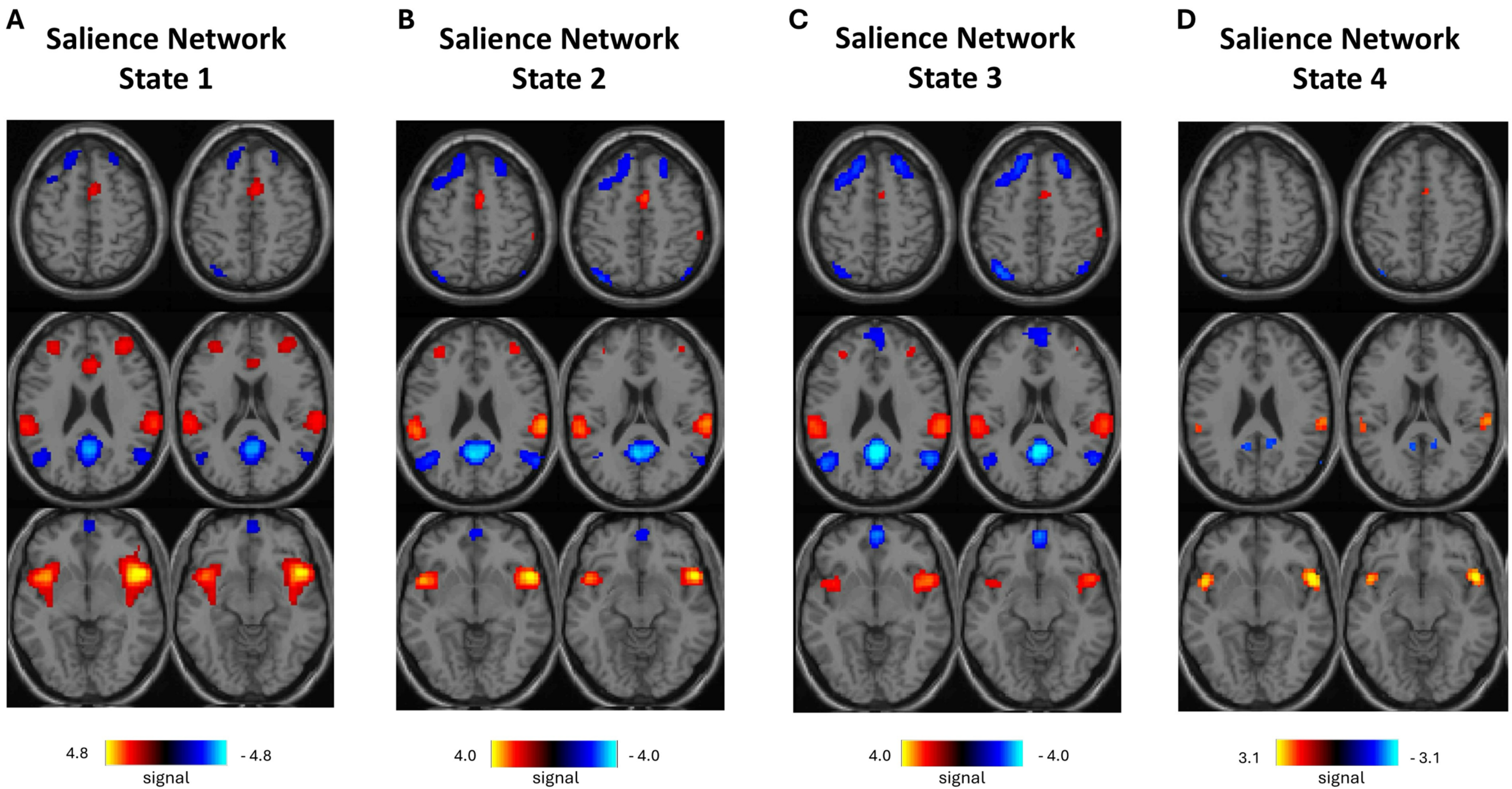

Functional imaging data were processed with GIFT toolbox (http://trendscenter.org/software/gift). We applied a spatially constrained multivariate objective optimization ICA with reference (MOO-ICAR) to extract 12 spatial maps of large-scale brain networks. For this study, we selected the Salience Network (SN) as our network of interest because primarily affected in FTD. The spatial chronnectome analysis was conducted using the dynamic FNC toolbox implemented in GIFT toolbox (http:// trendscenter.org/software/gift). For each subject, the dynamic component maps (dCMs) of SN were computed using a sliding window approach and then clustered via k-means into a discrete set of spatial states. The number of spatial states was set to 4. Specifically, k -means clustering was applied on the 76,788 dCMs (711 subjects × 108 windows) of the brain network, and it was repeated 100 times with different initializations, as previously published. State-level dynamic metrics were calculated for the SN, in particular the mean dwell time (DT), that is the average of the amount of time that subjects spend in a state before transitioning, and the number of transitions (NT), that is the total number of transitions among SN states performed by subjects. The four spatial states (A,B,C,D) of the SN were characterized by a frontal hub, with a more pronounced signal in the insula region, bilaterally. State 1, 2, and 3 showed an additional anticorrelation with frontoparietal and temporal regions. (HC=280; Presymptomatic=325; Symptomatic=106)

Supplementary Figure 2. Sex, educational attainment and country of residence moderate the relationship between stage and clinical outcomes in autosomal dominant FTD mutation carriers.

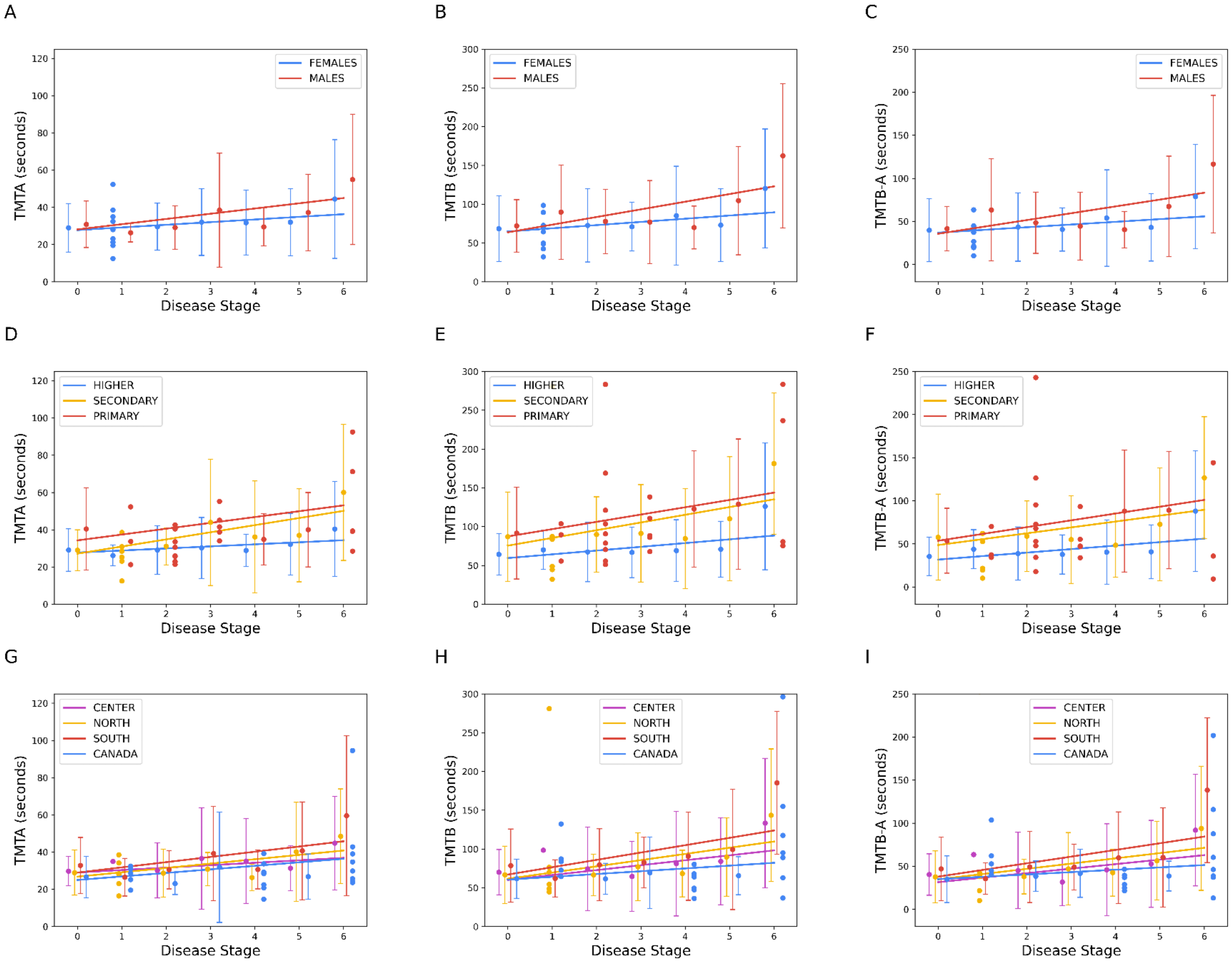

A-B-C= Results of the ANCOVA between biological disease stage and clinical outcome (TMT A, TMT B and TMT B-A scores). Female participants showed a significantly attenuated association between disease stage and clinical performance, as measured by TMT B, compared to males ($p<0.001$). This sex-related difference increased progressively across disease stages, with no difference observed at stage 0 and the largest divergences occurring at stages 5 and 6 (**panel B**). Comparable patterns were observed when cognitive performances were measured with TMT A (**panel A**) or the difference score TMT B-A (**panel C**). (N=711)

D-E-F= Results of the ANCOVA between biological disease stage and clinical outcome (TMT A, TMT B and TMT B-A scores). Individuals with higher education showed a weaker association between disease stage and cognitive decline than those with primary or secondary education ($p<0.001$). This effect was evident from stage 0 and increased steadily up to stage 6 (**panel E**). Similar findings were replicated when cognitive performances were measured by TMT A (**panel D**) or TMT B-A (**panel F**). (N=711)

G-H-I= Results of the ANCOVA between biological disease stage and clinical outcome (TMT A, TMT B and TMT B-A scores). Country of residence had a modest but significant effect, with a weaker stage-performance association observed in participants residing in Canada and Central Europe ($p=0.04$) (**panel H**). Again, similar results were found when using TMT A (**panel G**) and TMT B–A (**panel I**). (N=707)

TMT A = Trail Making Test part A; TMT B = Trail Making Test part B; TMT B-A = difference between TMT A results and TMT B results.
Circular dots represent the class average at a specific stage, bands represent standard deviation. If less than 10 participants are available at a specific stage standard deviation is not shown. Panels A, B, C group numerosity: Females = [stage0: 93, stage1: 9, stage2: 84, stage3: 67, stage4: 46, stage5: 70, stage6: 30]; Males = [stage0: 93, stage1: 13, stage2: 46, stage3: 54, stage4: 23, stage5: 46, stage6: 37]. Panels D, E, F group numerosity: Higher = [stage0: 137, stage1: 13, stage2: 99, stage3: 87, stage4: 47, stage5: 78, stage6: 40]: Secondary = [stage0: 35, stage1: 6, stage2: 22, stage3: 28, stage4: 11, stage5: 25, stage6: 20]; primary = [stage0: 14, stage1: 3, stage2: 9, stage3: 6, stage4: 11, stage5: 13, stage6: 7]. Panels G, H, I group numerosity: Center = [stage0: 25, stage1: 1, stage2: 44, stage3: 32, stage4: 22, stage5: 59, stage6: 20]; North = [stage0: 55, stage1: 6, stage2: 20, stage3: 29, stage4: 13, stage5: 13, stage6: 16]; South = [stage0: 66, stage1: 10, stage2: 51, stage3: 33, stage4: 27, stage5: 29, stage6: 20]; Canada = [stage0: 40, stage1: 5, stage2: 13, stage3: 27, stage4: 7, stage5: 15, stage6: 9]. Results of ANCOVA tests: Panel A: $F=7.44$, $p=0.0065$; Panel B: $F=11.6$, $p<0.001$; Panel C: $F=10.8$, $p=0.0011$; Panel D: $F=16.3$, $p<0.001$; Panel E: $F=29.2$, $p<0.001$; Panel F: $F=27.4$, $p<0.001$; Panel G: $F=3.22$, $p=0.022$; Panel H: $F=4.55$, $p=0.0036$; Panel I: $F=3.98$, $p=0.079$.

Supplementary Figure 3. P-values of Chi-squared test (A) and Cramér's V correlations (B) to quantify the statistical significance and effect size of the modulator interactions.

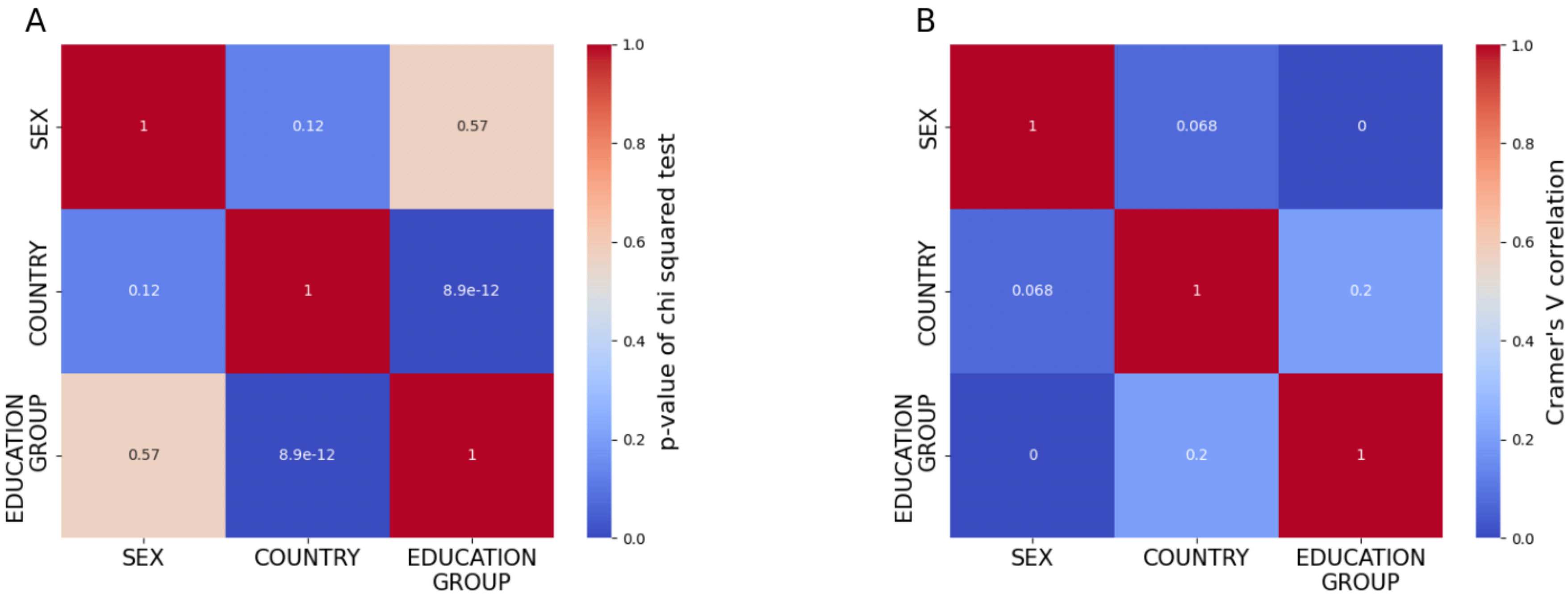

Statistical tests sample sizes: gender vs country N = 707 (N Females = 399, N Males = 312), gender vs education_group N=711 (N Higher = 501, N Secondary = 147, N Primary = 63), country vs education_group N=707 (N Center = 203, N North = 152, N South = 236, N Canada = 116).

GENFI Consortium members and affiliations.

- Rhian Convery, Department of Neurodegenerative Disease, Dementia Research Centre, UCL Queen Square Institute of Neurology, WC1N 3BG, London, UK
- Martina Bocchetta, Department of Neurodegenerative Disease, Dementia Research Centre, UCL Queen Square Institute of Neurology, WC1N 3BG, London, UK
- David Cash, Department of Neurodegenerative Disease, Dementia Research Centre, UCL Queen Square Institute of Neurology, WC1N 3BG, London, UK
- Sophie Goldsmith, Department of Neurodegenerative Disease, Dementia Research Centre, UCL Queen Square Institute of Neurology, WC1N 3BG, London, UK
- Kiran Samra, Department of Neurodegenerative Disease, Dementia Research Centre, UCL Queen Square Institute of Neurology, WC1N 3BG, London, UK
- David L. Thomas, Neuroimaging Analysis Centre, Department of Brain Repair and Rehabilitation, UCL Institute of Neurology, Queen Square, WC1N 3BG, London, UK
- Thomas Cope, Cambridge University Hospitals NHS Trust, University of Cambridge, CB2 0QQ, Cambridge, UK
- Maura Malpetti, Department of Clinical Neurosciences, University of Cambridge, CB2 1TN, Cambridge, UK
- Antonella Alberici, Centre for Neurodegenerative Disorders, Department of Clinical and Experimental Sciences, University of Brescia, Brescia, Italy
- Emanuele Buratti, ICGEB Trieste, Italy
- Andrea Arighi, Fondazione Ca' Granda, IRCCS Ospedale Maggiore Policlinico, 20122, Milan, Italy

- Chiara Fenoglio, Department of Biomedical, Surgical and Oral Sciences, University of Milan, Centro Dino Ferrari, Milan, Italy; Fondazione IRCCS Ca' Granda, Ospedale Maggiore Policlinico, Neurodegenerative Diseases Unit, 20122, Milan, Italy
- Vittoria Borracci, Fondazione IRCCS Ca' Granda, Ospedale Maggiore Policlinico, Neurodegenerative Diseases Unit, 20122, Milan, Italy
- Maria Serpente, Fondazione IRCCS Ca' Granda, Ospedale Maggiore Policlinico, Neurodegenerative Diseases Unit, 20122, Milan, Italy
- Tiziana Carandini, Fondazione IRCCS Ca' Granda, Ospedale Maggiore Policlinico, Neurodegenerative Diseases Unit, 20122, Milan, Italy
- Emanuela Rotondo, Fondazione IRCCS Ca' Granda, Ospedale Maggiore Policlinico, Neurodegenerative Diseases Unit, 20122, Milan, Italy
- Giacomina Rossi, Fondazione IRCCS Istituto Neurologico Carlo Besta, 20126, Milano, Italy
- Giorgio Giaccone, Fondazione IRCCS Istituto Neurologico Carlo Besta, 20126, Milano, Italy
- Pietro Tiraboschi, Fondazione IRCCS Istituto Neurologico Carlo Besta, 20126, Milano, Italy
- Paola Caroppo, Fondazione IRCCS Istituto Neurologico Carlo Besta, 20126, Milano, Italy
- Sara Prioni, Fondazione IRCCS Istituto Neurologico Carlo Besta, 20126, Milano, Italy
- Veronica Redaelli, Fondazione IRCCS Istituto Neurologico Carlo Besta, 20126, Milano, Italy
- David Tang-Wai, The University Health Network, Krembil Research Institute, M5T 2S8, Toronto, Canada
- Ekaterina Rogaeva, Tanz Centre for Research in Neurodegenerative Diseases, University of Toronto, M5T 0S8, Toronto, Canada
- Miguel Castelo-Branco, Faculty of Medicine, ICNAS, CIBIT, University of Coimbra, 3000-370, Coimbra, Portugal
- Morris Freedman, Baycrest Health Sciences, Rotman Research Institute, University of Toronto, M6A 2E1, Toronto, Canada
- Ron Keren, The University Health Network, Toronto Rehabilitation Institute, M5G 2A2, Toronto, Canada
- Sandra Black, Sunnybrook Health Sciences Centre, Sunnybrook Research Institute, University of Toronto, M4N 3M5, Toronto, Canada

- Sara Mitchell, Sunnybrook Health Sciences Centre, Sunnybrook Research Institute, University of Toronto, M4N 3M5, Toronto, Canada
- Christen Shoesmith, Department of Clinical Neurological Sciences, University of Western Ontario, N6A 5A5, London, Ontario, Canada
- Robert Bartha, Department of Medical Biophysics, The University of Western Ontario, N6A 5C1, London, Ontario, Canada; Centre for Functional and Metabolic Mapping, Robarts Research Institute, The University of Western Ontario, London, Ontario, Canada
- Rosa Rademakers, Center for Molecular Neurology, University of Antwerp, B-2610, Antwerp, Belgium
- Jackie Poos, Department of Neurology, Erasmus Medical Center, 3000 CA, Rotterdam, Netherlands
- Janne M. Papma, Department of Neurology, Erasmus Medical Center, 3000 CA, Rotterdam, Netherlands
- Lucia Giannini, Department of Neurology, Erasmus Medical Center, 3000 CA, Rotterdam, Netherlands
- Liset de Boer, Department of Neurology, Erasmus Medical Center, 3000 CA, Rotterdam, Netherlands
- Julie de Houwer, Department of Neurology, Erasmus Medical Center, 3000 CA, Rotterdam, Netherlands
- Rick van Minkelen, Department of Clinical Genetics, Erasmus Medical Center, Rotterdam, Netherlands
- Yolande Pijnenburg, Amsterdam University Medical Centre, Amsterdam VUMC, Amsterdam, Netherlands
- Camilla Ferrari, Department of Neuroscience, Psychology, Drug Research and Child Health, University of Florence, 50139, Florence, Italy
- Cristina Polito, Department of Biomedical, Experimental and Clinical Sciences “Mario Serio”, Nuclear Medicine Unit, University of Florence, 50134, Florence, Italy
- Gemma Lombardi, Department of Neuroscience, Psychology, Drug Research and Child Health, University of Florence, 50139, Florence, Italy
- Valentina Bessi, Department of Neuroscience, Psychology, Drug Research and Child Health, University of Florence, 50139, Florence, Italy
- Enrico Fainardi, Neuroradiology Unit, Department of Experimental and Clinical Biomedical Sciences, University of Florence, 50134, Florence, Italy
- Stefano Chiti, Neuroradiology Unit, Department of Experimental and Clinical Biomedical Sciences, University of Florence, Florence, Italy.

- Mattias Nilsson, Department of Clinical Neuroscience, Karolinska Institutet, 171 77, Stockholm, Sweden
- Henrik Viklund, Karolinska University Hospital Huddinge, 141 86, Sweden
- Melissa Taheri Rydell, Department of Neurobiology, Care Sciences and Society, Center for Alzheimer Research, Division of Neurogeriatrics, Bioclinicum, Karolinska Institutet, 171 64, Solna, Sweden; Unit for Hereditary Dementias, Theme inflammation and Aging, Karolinska University Hospital, Solna, Sweden
- Vesna Jelic, Department of Neurobiology, Care Sciences and Society; Division of Clinical Geriatrics, Karolinska Institutet, Stockholm, Sweden; Cognitive clinic, Theme inflammation and Aging, Karolinska University Hospital, Solna, Sweden
- Abbe Ullgren, Department of Neurobiology, Care Sciences and Society,; Center for Alzheimer Research, Division of Neurogeriatrics, Bioclinicum, Karolinska Institutet, Solna, Sweden; Unit for Hereditary Dementias Theme inflammation and Aging, Karolinska University Hospital, SE-171 76, Solna, Sweden
- Elena Rodriguez-Vieitez, Department of Neurobiology, Care Sciences and Society, Center for Alzheimer Research, Division of Neurogeriatrics, Bioclinicum, Karolinska Institutet, 171 64, Solna, Sweden; Unit for Hereditary Dementias, Theme Inflammation and Aging, Karolinska University Hospital, Solna, Sweden
- Tobias Langheinrich, Division of Neuroscience and Experimental Psychology, Wolfson Molecular Imaging Centre, University of Manchester, M13 9PL, Manchester, UK; Manchester Centre for Clinical Neurosciences, Department of Neurology, Salford Royal NHS Foundation Trust, Manchester, UK
- Albert Lladó, Alzheimer’s disease and Other Cognitive Disorders Unit, Neurology Service, Hospital Clínic, 170 08036, Barcelona, Spain
- Anna Antonell, Alzheimer’s disease and Other Cognitive Disorders Unit, Neurology Service, Hospital Clínic, 170 08036, Barcelona, Spain
- Jaume Olives, Alzheimer’s disease and Other Cognitive Disorders Unit, Neurology Service, Hospital Clínic, 170 08036, Barcelona, Spain
- Mircea Balasa, Alzheimer’s disease and Other Cognitive Disorders Unit, Neurology Service, Hospital Clínic, 170 08036, Barcelona, Spain

- Nuria Bargalló, Imaging Diagnostic Center, Hospital Clínic, 170 08036, Barcelona, Spain
- Sergi Borrego-Ecija, Alzheimer's disease and Other Cognitive Disorders Unit, Neurology Service, Hospital Clínic, 170 08036, Barcelona, Spain
- Ana Verdelho, Department of Neurosciences and Mental Health, Centro Hospitalar Lisboa Norte Hospital de Santa Maria & Faculty of Medicine, University of Lisbon, 1649-028, Lisbon, Portugal
- Carolina Maruta, Laboratory of Language Research, Centro de Estudos Egas Moniz, Faculty of Medicine, University of Lisbon, 1500-650, Lisbon, Portugal
- Tiago Costa-Coelho, Faculty of Medicine, University of Lisbon, 1649-028, Lisbon, Portugal; Institute of Molecular Medicine João Lobo Antunes, University of Lisbon, Lisbon, Portugal; Research Institute for Medicines, Faculty of Pharmacy, University of Lisbon, Lisbon, Portugal
- Gabriel Miltenberger, Faculty of Medicine, University of Lisbon, 1649-028, Lisbon, Portugal
- Frederico Simões do Couto, Faculdade de Medicina, Universidade Catolica Portuguesa 1649-023, Lisbon, Portugal
- Alazne Gabilondo, Cognitive Disorders Unit, Department of Neurology, Donostia University Hospital, 20014**,** San Sebastian, Gipuzkoa, Spain; Instituto de Investigación Sanitaria Biogipuzkoa, Neurosciences Area, Group of Neurodegenerative Diseases, San Sebastian, Spain; Center for Biomedical Research in Neurodegenerative Disease (CIBERNED), Carlos III Health Institute, Madrid, Spain
- Ioana Croitoru, Instituto de Investigacion Sanitaria Biogipuzkoa, Neurosciences Area, Group of Neurodegenerative Diseases, 20014, San Sebastian, Spain; Center for Biomedical Research in Neurodegenerative Disease (CIBERNED), Carlos III Health Institute, Madrid, Spain
- Mikel Tainta, Instituto de Investigacion Sanitaria Biogipuzkoa, Neurosciences Area, Group of Neurodegenerative Diseases, 20014, San Sebastian, Spain; Center for Biomedical Research in Neurodegenerative Disease (CIBERNED), Carlos III Health Institute, Madrid, Spain

- Myriam Barandiaran, Cognitive Disorders Unit, Department of Neurology, Donostia University Hospital, 20014**,** San Sebastian, Gipuzkoa, Spain; Instituto de Investigación Sanitaria Biogipuzkoa, Neurosciences Area, Group of Neurodegenerative Diseases, San Sebastian, Spain¸Center for Biomedical Research in Neurodegenerative Disease (CIBERNED), Carlos III Health Institute, Madrid, Spain
- Patricia Alves, Instituto de Investigacion Sanitaria Biogipuzkoa, Neurosciences Area, Group of Neurodegenerative Diseases, 20014, San Sebastian, Spain; Department of Educational Psychology and Psychobiology, Faculty of Education, International University of La Rioja, Logroño, Spain
- Benjamin Bender, Department of Diagnostic and Interventional Neuroradiology, University of Tübingen, 72076, Tübingen, Germany
- David Mengel, Department of Neurodegenerative Diseases, Hertie Institute for Clinical Brain Research and Center of Neurology, University of Tübingen, 72076 , Tübingen, Germany; Center for Neurodegenerative Diseases (DZNE), Tübingen, Germany
- Lisa Graf, Department of Neurodegenerative Diseases, Hertie Institute for Clinical Brain Research and Center of Neurology, University of Tübingen, 72076 , Tübingen, Germany
- Annick Vogels, Department of Human Genetics, KU Leuven, 3000, Leuven, Belgium
- Mathieu Vandenbulcke, Geriatric Psychiatry Service, University Hospitals Leuven, Belgium; Neuropsychiatry, Department of Neurosciences, KU Leuven, 3000, Leuven, Belgium
- Philip Van Damme, Neurology Service, University Hospitals Leuven, Belgium; Laboratory for Neurobiology, VIB-KU Leuven Centre for Brain Research, 3000, Leuven, Belgium
- Rose Bruffaerts, Department of Biomedical Sciences, University of Antwerp, 2610, Antwerp, Belgium; Biomedical Research Institute, Hasselt University, 3500, Hasselt, Belgium
- Koen Poesen, Laboratory for Molecular Neurobiomarker Research, KULeuven, 3000, Leuven, Belgium
- Pedro Rosa-Neto, Translational Neuroimaging Laboratory, McGill Centre for Studies in Aging, McGill University, H4H 1R3 , Montreal, Québec, Canada

- Maxime Montembeault, Douglas Research Centre, Department of Psychiatry, McGill University, H4H 1R3, Montreal, Québec, Canada
- Raffaella Lara Migliaccio, Sorbonne Université, Paris Brain Institute – Institut du Cerveau – ICM, Inserm U1127, CNRS UMR 7225, AP-HP - Hôpital Pitié-Salpêtrière, Paris, France; Centre de référence des démences rares ou précoces, IM2A, Département de Neurologie, AP-HP - Hôpital Pitié-Salpêtrière, Paris, France;
- Ninon Burgos, Sorbonne Université, Paris Brain Institute–Institut du Cerveau– ICM, Inserm U1127, CNRS UMR 7225, AP-HP Hôpital Pitié-Salpêtrière, 75646, Paris, France, Paris, France; Inria, Aramis project-team, F-75013, Paris, France; Centre pour l'Acquisition et le Traitement des Images, Institut du Cerveau et la Moelle, Paris, France
- Daisy Rinaldi, Sorbonne Université, Paris Brain Institute–Institut du Cerveau– ICM, Inserm U1127, CNRS UMR 7225, AP-HP Hôpital Pitié-Salpêtrière, 75646, Paris, France, Paris, France; Centre de référence des démences rares ou précoces, IM2A, Département de Neurologie, AP-HP - Hôpital Pitié-Salpêtrière, Paris, France; Département de Neurologie, AP-HP - Hôpital Pitié-Salpêtrière, Paris, France
- Catharina Prix, Neurologische Klinik, Ludwig-Maximilians-Universität München, 81377, Munich, Germany
- Elisabeth Wlasich, Neurologische Klinik, Ludwig-Maximilians-Universität München, 81377, Munich, Germany
- Olivia Wagemann, Neurologische Klinik, Ludwig-Maximilians-Universität München, 81377, Munich, Germany
- Sonja Schönecker, Neurologische Klinik, Ludwig-Maximilians-Universität München, 81377, Munich, Germany
- Alexander Maximilian Bernhardt, Neurologische Klinik, Ludwig-Maximilians-Universität München, 81377, Munich, Germany
- Anna Stockbauer, Neurologische Klinik, Ludwig-Maximilians-Universität München, 81377, Munich, Germany
- Jolina Lombardi, Department of Neurology, University of Ulm, 89081, Ulm, Germany
- Sarah Anderl-Straub, Department of Neurology, University of Ulm, 89081, Ulm, Germany
- Adeline Rollin, CHU, CNR-MAJ, Labex Distalz, LiCEND, 59037, Lille, France

- Gregory Kuchcinski, Univ Lille, France; Inserm 1172, Lille, France; CHU, CNR-MAJ, Labex Distalz, LiCEND, 59037, Lille, France
- Vincent Deramecourt, Univ Lille, France; Inserm 1172, Lille, France; CHU, CNR-MAJ, Labex Distalz, LiCEND, 59037, Lille, France
- João Durães, Neurology Department, Centro Hospitalare Universitario de Coimbra, 3004-561, Coimbra, Portugal
- Marisa Lima, Neurology Department, Centro Hospitalare Universitario de Coimbra, 3004-561, Coimbra, Portugal
- Maria João Leitão, Centre of Neurosciences and Cell Biology, Universidade de Coimbra, 3004-517, Coimbra, Portugal
- Maria Rosario Almeida, Faculty of Medicine, University of Coimbra, 3000-370, Coimbra, Portugal
- Miguel Tábuas-Pereira, Neurology Department, Centro Hospitalar e Universitario de Coimbra, Coimbra, Portugal; Faculty of Medicine, University of Coimbra, 3000-370, Coimbra, Portugal
- Sónia Afonso, Instituto Ciencias Nucleares Aplicadas a Saude, Universidade de Coimbra, 3000-548, Coimbra, Portugal
- João Lemos, Faculty of Medicine, University of Coimbra, 3000-370, Coimbra, Portugal
